# Psychosocial factors predicting risky sexual behaviour among long distance truck drivers in Lagos, Nigeria

**DOI:** 10.1080/17290376.2017.1405838

**Published:** 2017-11-27

**Authors:** Abiodun Musbau Lawal, Benjamin Oladapo Olley

**Affiliations:** ^a^ BSc Psychology, MSc Clinical Psychology & PhD Clinical Psychology, Lecturer I, Department of Psychology, Federal University Oye-Ekiti, Oye-Ekiti, Nigeria; ^b^ BSc Psychology, MSc Clinical Psychology & PhD Clinical Psychology, Associate Professor, Department of Psychology, University of Ibadan, Ibadan, Nigeria

**Keywords:** risky sexual behaviour, psychological factors, social factors, long distance truck drivers, Nigeria

## Abstract

Long distance truck drivers (LDTDs) have been identified as one of the groups at higher risk for human immunodeficiency virus (HIV) infection. Understanding how certain social and psychological variables that have a strong theoretical basis contribute to sexual risk behaviour will guide in the implementation process of HIV risk-reduction intervention in the trucking population. In line with the conceptualisation of Information, Motivation and Behavioural skills model, we examined the extent that HIV knowledge, attitude towards condom use, peer support to condom use, perceived vulnerability to HIV/AIDS, and condom use self-efficacy will independently and jointly explain sexual risk behaviours of LDTDs in a haulage company in Lagos, Nigeria. A cross-sectional survey design was used and 154 drivers with ages ranging from 27 to 68 years (*M* = 44.03, SD = 8.82) completed copies of a questionnaire comprising demographics and measures of psychological variables. Psychological factors that included HIV knowledge, attitude towards condom use, perceived vulnerability to HIV/AIDS, peer support to condom use, and condom use self-efficacy significantly jointly predicted sexual risk behaviours (*R* ^2^ = .59, *F*(5, 148) = 42.63; *p* < .05), by accounting for about 59% of the explained variance in sexual risk behaviours. Social factors that included age, number of years of education, number of wives, number of intercourses in the last three months, number of partners apart from primary partners, and number of weeks spent outside home significantly jointly predicted sexual risk behaviour (*R* ^2^ = .15, *F*(6, 147) = 4.39; *p* < .05) by accounting for about 15% of the explained variance in sexual risk behaviour among the drivers. It is concluded that all the psychological and social factors examined as predictor variables could jointly play important roles in prevention intervention programmes for reducing sexual risk behaviours of LDTDs. Stakeholders should sensitise LDTDs on the need to realise that they are a high-risk group and are more vulnerable to HIV infection; thus, behaviour change is indispensable in their sexual relationships.

## Introduction

Sexual risk behaviours seem to be a major route through which human immunodeficiency virus (HIV) as well as other sexually transmitted infections (STIs) are spread within different populations. Long distance truck drivers (LDTDs) have been reported as one of the groups at higher risk for HIV infection throughout the world (Atilola, Akpa, & Komolafe, 2010; Azuonwu, Erhabor, & Frank-Peterside, 2011; Delany-Moretlwe et al., 2014; Ishtiaq, Asif, Jamil, Irfan, & Ishtiaq, 2017; Maarefvand, Kassaie, Ghiasvand, Abolfathi Gharibdoosti, & Khubchandani, 2016; Sawal, Hans, & Verma, 2016; Singh & Joshi, 2012). This assertion is based on drivers’ involvement in various sexual risk behaviours, which include unprotected sex, casual sexual relationship, heavy use of alcohol before sex, and frequent unprotected sex with commercial sex workers (CSW) (Aral & Holmes, 2006; Matovu & Ssebadduka, 2014; Pandey et al., 2008; Sawal et al., 2016; Sunmola, 2005).

The level of LDTDs’ involvement in sexual risk behaviours may be reduced if effective HIV prevention intervention that has a strong theoretical basis is employed. This conforms to Fisher and Fisher’s (1992) assertion that HIV prevention intervention that has Information, Motivation and Behavioural skills (IMB) components would be more appropriate in reducing sexual risk behaviour. The IMB model proposes that information which is relevant to preventive behaviour, motivation to act on this information, and behavioural skills for acting on this information effectively are fundamental determinants of preventive behaviour. Relating the model to this study, HIV prevention knowledge, motivation to use condoms, social support to the use of condoms, and seeing oneself as less vulnerable to HIV infection activates a driver to regularly and consistently use condoms in their sexual relationships. Thus, certain psychological variables that are related to the IMB skills model need to be considered in HIV prevention and intervention programmes (Lawal & Olley, 2015). With this in mind, examining HIV knowledge as a related variable to information component, attitude towards condoms, perceived vulnerability to HIV/AIDS, and peer support to condom use as motivation components, while condom use self-efficacy as the behavioural skill component of IMB model, is appropriate in this study.

Sexual risk behaviours have been commonly documented across different populations in Nigeria (Imaledo, Peter-Kio, & Asuquo, 2012; Odimegwu & Somefun, 2017), South Africa (Pengpid, Peltzer, & Skaal, 2013), and India (Sahu et al., 2014; Sunitha & Gururaj, 2014). Some of the various risky sexual behaviours documented include inconsistent and incorrect use of condoms, heavy alcohol use before sex, unprotected sex with CSWs, casual sex, and multiple sexual partners. These aforementioned sexual risk behaviours have also been reported as common among LDTDs worldwide (Matovu & Ssebadduka, 2014; Pandey et al., 2008). In Nigeria, however, the few available studies conducted on truck drivers confirm the prevalence of sexual risk behaviours such as low rate of condom use (Arulogun, Oladepo, & Titiloye, 2011; Sunmola, 2005) and the need to research on this population as a high-risk group to HIV/AIDS (Aniebue & Aniebue, 2011).

Sexual risk behaviour in the trucking population may have some underlying psychological and social factors. Psychological factors such as HIV knowledge, attitude towards condom use, peer support to condom use, perceived vulnerability to HIV/AIDS, and condom use self-efficacy may directly or jointly contribute to high levels of sexual risk behaviour among LDTDs. Similarly, social factors such as age, number of years of education, number of wives, number of intercourses in the last three months, number of partners apart from primary partners, and number of weeks spent outside home may have some relationships with sexual risk behaviour among the drivers.

Knowledge about the levels of HIV infection in a population, how HIV is transmitted, and how to avoid contracting it may contribute to how people who engage in high risky behaviour would adopt safe sexual behaviours or refrain from sexual risk behaviour all together. In Nigeria, HIV knowledge has been found to relate with sexual risk behaviour among the drivers (Atilola et al., 2010; Awosan, Ibrahim, Arisegi, & Erhiano, 2014; Olugbenga-Bello, Asekun-Olarinmoye, & Adeomi, 2011). Focusing on increasing HIV knowledge was said to be insufficient in itself in HIV prevention intervention strategies (Rugigana, Birungi, & Nzayirambaho, 2015). Therefore, having the knowledge of HIV/AIDS may not be enough in the trucking population. An individual who is knowledgeable about HIV infection may also require other relevant factors that would help in reducing any form of sexual risk behaviour. In other words, being knowledgeable about HIV and AIDS may not be enough to ensure a change in risky sexual behaviour among LDTDs.

Condom use has been reported to be highly effective against contacting HIV and other STIs, when it is used correctly and consistently (Centers for Disease Control and Prevention, 2006; UNAIDS, 2014). Therefore, attitude towards condom use is very germane in explaining safe sexual behaviour among individuals. On this note, attitude towards condom use may contribute to sexual risk behaviour among LDTDs. Previous studies have shown that LDTDs with a positive attitude towards condom use were more likely to use condoms when the need arises (Atilola et al., 2010; Matovu & Ssebadduka, 2014; Sorensen, Anderson, Speaker, Menacho, & Vilches, 2007).

Social support is critical to an individual's personal change because changes take place within a network of social influences. People within a given social network can either support or neglect various behaviours associated with sexual practices. In support of this assertion is Bandura’s (1994) report that ‘behaviour that violates prevailing social norms brings social censure or other punishing consequences; whereas behaviour that fulfills socially valued norms is approved and rewarded’. Peer support to condom use can help a driver to adopt the use of condoms, and thus reduce his involvement in sexual risk behaviour. This suggests that when condom use is encouraged in a particular group, there is a higher tendency for a group member to have a positive attitude towards condom use.

Another factor that may contribute to explaining sexual risk behaviour of LDTDs is a person’s ability to negotiate condom use with the partner, known as condom use self-efficacy. O’Leary, Jemmott, and Jemmott (2008) highlighted the importance of self-efficacy in explaining the effects of skill-building sexual risk-reduction interventions on sexual risk behaviour. Similarly, Lawal and Olley (2015) stressed the importance of condom use self-efficacy in behavioural skill training for LDTDs. Condom use self-efficacy in this context is the drivers’ ability to effectively negotiate the use of condoms with sexual partners and regularly make use of condoms during sex.

Perceived vulnerability to HIV/AIDS is another variable that needs to be included in the group of psychological factors that could help in explaining sexual risk behaviour of LDTDs. Self-perception of risk is an individual's belief that he or she will be able to contract certain disease. Previous studies have shown a connection between perceived vulnerability to HIV/AIDS and sexual risk behaviour in the trucking population (Atilola et al., 2010; Magno & Castellanos, 2016). In their study conducted in Nigeria, Atilola et al. (2010) reported 68.1% drivers admitted to being vulnerable to HIV infection. Therefore, LDTDs’ perceptions of personal vulnerability to HIV/AIDS may be the reflections of current and recent sexual risk behaviours, and this may help in explaining their sexual risk behaviour.

Some living and working conditions put LDTDs at risk of contracting and transmitting HIV infection. International Organisation for Migration (IOM) identified the following working conditions as contributing to risk of contracting HIV infection among LDTDs: being separated from their regular partners for extended periods of time, subjected to stress, attractive customers to CSW who are readily available at the so-called hot spots where trucks stop, and drivers often have inadequate access to health services that include treatment for STIs (IOM, 2005; Tiang et al., 2010). To a large extent, these conditions account for a higher HIV prevalence among the trucking population (IOM, 2012). This suggests that certain social factors associated with drivers’ working conditions might have some levels of contributions in the extent to which LDTDs engage in sexual risk behaviours.

Soldan, deGraft, Bisika, and Tsiu (2007) evidenced that some socio-demographic and economic variables are determinants of risky sexual behaviours such as having ever paid for sex, total number of sex partners in the past year, and having ever used condoms. In the current study, among the social factors examined to influence sexual risk behaviour of drivers were age and education. Ages of individuals have been developmentally linked to sexual risk behaviours (Bachanas et al., 2002). Also, some previous studies on LDTDs reported an association between age and forms of sexual risk behaviours (Awosan et al., 2014; Bhatnagar, Sakthivel Saravanamurthy, & Detels, 2015; Yaya et al., 2016; Zahiruddin, Gaidhane, Shanbhag, & Zodpey, 2011). Maarefvand et al. (2016) but, however, noted that the regular use of condoms was statistically significantly higher in younger drivers than older ones. In terms of linking education and risky sexual behaviour, Yaya et al. (2016) reported that risk drivers with a high level of education consistently used condoms. This perhaps suggests that education enlightens understanding of HIV preventive information provided as a form of risk-reduction interventions among the drivers. Also, having multiple sexual partners can predispose drivers to higher engagement in sexual risk behaviour. In line with previous studies (Kohli et al., 2017; Sastry, 2016) that have reported structural or working conditions as contributors to sexual behaviour of truck drivers, we included the number of weeks the drivers spent outside their homes as a possible influencing social factor.

## Objective of the study

The main purpose of this study was to examine the extent of the direct and joint contribution of HIV knowledge, attitude towards condom use, peer support to condom use, perceived vulnerability to HIV/AIDS, and condom use self-efficacy in explaining sexual risk behaviours of LDTDs in a haulage company in Lagos state. Also, to examine age, number of years of education, number of wives, number of intercourses in the last three months, number of partners apart from primary partners, and number of weeks spent outside home as social predictors of sexual risk behaviour among the drivers.

## Statement of hypotheses

1. HIV knowledge, attitude towards condom use, perceived vulnerability to HIV/AIDS, perceived peer support to condom use, and condom use self-efficacy will independently and jointly significantly predict sexual risk behaviour of LDTDs.
2. Age, number of years of education, number of wives, number of intercourses in the last three months, number of partners apart from primary partners, and number of weeks spent outside home will independently and jointly significantly predict the sexual risk behaviour of LDTDs.

## Methods

### Research design

A cross-sectional survey design was employed with the use of questionnaires that were administered in order to determine the relationships among variables of interest in the study. The study was cross-sectional in approach because the study's data were collected from the participants at once. In the study, independent variables were HIV knowledge, attitude towards condom use, perception of vulnerability to HIV/AIDS, condom use self-efficacy, and peer support to the use of condoms. Sexual risk behaviour is the outcome variable.

### Participants

One hundred and fifty-four drivers were selected with the use of accidental sampling technique from the total population of 250 LDTDs in a corporate organisation in Lagos state. We used Slovin’s formula to calculate the sample size (*n*), given the population size (*N*) and a margin of error (*e*). It was computed as *n* = *N*/(1 + *Ne* ^2^). The formula gives the researcher an idea of how large his sample size needs to be to ensure a reasonable accuracy of results (Ariola Mariano, 2006). With a population of 250 LDTDs in the haulage company and margin error of 0.05, the researchers plugged in these data into the formula to arrive at 154 as the sample size for the survey. Using Slovin’s formula, the researchers arrived at the sample size as follows: 250/(1 + (250 × (0.05 × 0.05)) = 250/(1 + (250 × 0.0025)) = 250/(1 + 0.625) = 250/1.625 = 153.85 = 154. The final result was 154 which was the number of LDTDs sampled for the study.

The LDTDs’ mean age was 44.03 years with a standard deviation of 8.82 years and age range of 27–68 years. Analysis of number of sexual encounters in three months prior to the data collection period showed a minimum of 1 to a maximum of 22 times/episodes. The participants had 0–4 wives, and the number of sexual partners apart from primary partners ranged from 0 to 13 women. LDTDs spent a maximum of 13 weeks outside home within three months prior to the current study with an average of 3.97 weeks. Distribution of other demographical characteristics of drivers is presented in Table 1.

**Table 1.** Socio-demographic characteristics of drivers.

Variable	*N*	Percentage
*Religion*		
Christianity	44	28.6
Islam	88	57.1
Others	10	6.5
No religion	12	7.8
*Educational qualification*		
No formal education	40	26
Primary education	55	35.7
Secondary education	54	35.1
First degree	5	3.2
*Alcohol use*		
Yes	128	83.1
No	26	16.9
*Tobacco use*		
Yes	97	63
No	57	37

#### Measures

We used a structured questionnaire as an instrument for data collection in the study. The questionnaire consisted of socio-demographic variables and standardised scales with acceptable psychometric properties. The scales included in the questionnaire were as follows:

HIV knowledge was assessed using a 22-item HIV knowledge (HIV) modified version of the original 18-item HIV knowledge scale developed by Carey and Schroder (2002). Items in the scale include ‘Coughing and sneezing DO NOT spread HIV’ to ‘A person can get HIV from oral sex’. On the scale, respondents with a mean score of 9.94 and above have higher HIV knowledge while those with a score below the mean score have lower HIV knowledge. A Cronbach's alpha of 0.75 was reported in the study.

Perceived vulnerability to HIV/AIDS was assessed with the use of 7-item perceived vulnerability to HIV/AIDS scale adapted from the work of Koopman and Reid (1998). The scale measures drivers’ perception of risk in contracting HIV and AIDS. Examples of the test items include ‘I am at risk for HIV/AIDS’ and ‘There is a possibility that I have HIV/AIDS.’ The scale is rated on a 5-point Likert-type response format, ranging from strongly disagree (scored 1) to strongly agree (scored 5). On the scale, respondents with a mean score of 23.43 and above have higher vulnerability to HIV/AIDS, while those with a score below the mean score have lower vulnerability to HIV/AIDS. We obtained Cronbach's alpha coefficient of 0.59 for the scale in the current study.

Perceived peer support to condom used was measured using the 5-item peer support to condom use scale adapted from the work of Moore (2008). The scale measures how peers of the drivers support the use of condoms. Examples of items in the scale include ‘My friends/significant others think condoms should be worn during sex’ and ‘My peers (i.e. drivers) think condoms should be used during sex’. The scale is rated on a 5-point Likert-type response format ranging from strongly disagree (scored 1) to strongly agree (scored 5). Respondents with a mean score of 14.32 and above on the scale have higher peer support to condom use, while those with a score below the mean score have lower peer support to condom use. In the current study, we reported a Cronbach's alpha reliability coefficient of 0.62 for the scale.

Condom use self-efficacy was assessed with the use of 28-item Condom use self-efficacy scale (CUSES) developed by Brafford and Beck (1991). The scale measures drivers’ expectations of success in obtaining, disposing of, and negotiating the use of condoms. Samples of items in the CUSES include ‘I feel confident in my ability to put a condom on myself or my partner’ and ‘I feel confident I could purchase condoms without feeling embarrassed’. The scale is rated on a 5-point Likert-type scale ranging from strongly disagree (scored 1) to strongly agree (scored 5). One the scale, respondents with a mean score of 66.38 and above have higher levels of ability to use condoms effectively while those with a score below the mean score have lower levels of ability to use condoms effectively. For this study, a Cronbach's alpha reliability coefficient of 0.81 was obtained for the scale.

Attitude towards condom use was measured using the 13-item Attitude towards condom use scale developed by DeHart and Birkimer (1997). Samples of items in the scale include ‘It is a hassle to use condoms’ and ‘People can get the same pleasure from “safer” sex as from unprotected sex’. The scale has 5-point Likert response format which ranged from strongly disagree (scored 1) to strongly agree (scored 5). On the scale, respondents with a mean score of 40.45 and above were regarded as having a positive attitude towards condom use, while those with a score below the mean score have a negative attitude towards condom use. A Cronbach's alpha reliability coefficient of 0.71 was obtained for the scale.

Sexual risk behaviour was measured using the 6-item Sexual Risk Behaviour Scale (SRBS) developed by Lawal (2013). The SRBS measures drivers’ levels of sexual risk behaviour in the last three months. Examples of items in the scale include ‘I have taken alcohol heavily before having sex in the last 3 months’ and ‘I have had sex with a casual friend I met for the first time in the last 3 months’. The scale has a 4-point Likert response format ranging from never (scored 1) to always (scored 4). None of the items in the scale was reverse-scored. On the scale, respondents with a mean score of 15.64 and above have higher sexual risk behaviour while those with a score below the mean score have lower sexual risk behaviour. We reported a Cronbach's alpha reliability coefficient of 0.85 for the scale.

### Sampling methods

Multi-stage sampling techniques were adopted in the study. Three companies were initially identified for the study considering the inclusion criteria, though only the one that fully met the inclusion criteria would be considered for the exercise. Of the three identified companies, only the haulage company fully met the inclusion criteria, especially on the basis of willingness to participate in the research exercise. The researchers, therefore, purposively identified the haulage company used among others in Lagos, Nigeria, having met the following inclusion criteria:

- Should have a population of LDTDs of more than 200.
- That movement of drivers in the corporate organisation is well determined in such a way that drivers could be converged in a place on their arrival from their trips.
- That a substantial number of drivers in the organisation are long distance drivers who are likely to be back in the office within a month.
- That the management of the organisation would be willing to participate in the exercise by giving its consent and all necessary assistance.

Furthermore, participants were selected for the study with the use of accidental sampling technique in the distribution of questionnaires. Those who consented to complete the questionnaires were eventually reached.

### Data collection procedure

Due to the sensitivity of the test items, we coordinated the administration of questionnaires by ourselves. However, the assistants who were provided by the management of the haulage company only helped in ensuring that the venue where the drivers converged to complete the questionnaires was conducive for data collection. The researchers administered questionnaires to the drivers within the premises of the organisation. The period of data collection took 12 weeks because of the mobility of drivers. With 50 drivers refusing consent, 200 questionnaires were distributed and 171 were returned. The number of questionnaires retrieved implies an 85.5% response rate of the total distributed. However, based on the sample size calculation, 154 questionnaires that were properly completed were used for data analysis in this study.

### Data analysis

The study data were entered and analysed using SPSS Statistics version 15. We computed both descriptive and inferential statistics in the study. Descriptive statistics such as frequencies, mean, standard deviation, and percentages were used to describe demographic characteristics of participants. Multiple regression as an inferential statistics was used to test the hypotheses in the study. We tested the relevant assumptions of the statistics on sample size and singularity of independent variables. First, with a sample size of 154, the study meets the assumption on sample size because a minimum of 96 participants was described suitable for including five predictor variables in multiple regression analysis (Tabachnick & Fidell, 2001). The current study also met singularity of independent variables because HIV knowledge, attitude towards condom use, perceived vulnerability of HIV/AIDS, peer support to condom use, and condom use self-efficacy were not a combination of other independent variables or one another. We then computed multiple regression analysis to determine the independent and joint contributions of predictor variables in explaining sexual risk behaviour of the drivers. All statistics in the analyses were significant at the 0.05 level of significance.

### Ethical consideration

Institutional-based ethical approval and permission to conduct the study were obtained from the Department of Psychology, University of Ibadan, Nigeria. Conduct of the research exercise was further approved by the management of the haulage company. This was done with several meetings with the management and the approval was given subsequently. Anonymity of participants and confidentiality of information the respondents provided were prime ethical issues that the researchers took earnestly. Verbal informed consent was initially obtained from every participant before they participated in the exercise, after which the drivers further established their consent by indicating ‘Yes’ on the questionnaires before completing them. Participation in the research exercise was voluntary and the participants were given the option of opting out at will.

## Results

### Hypotheses tested

*First hypothesis:* HIV knowledge, attitude towards condom use, perceived vulnerability to HIV/AIDS, perceived peer support to condom use, and condom use self-efficacy will independently and jointly significantly predict sexual risk behaviours among LDTDs. The result is presented in Table 2.

**Table 2.** Shows multiple regression of psychological factors predicting sexual risk behaviours of LDTDs.

Model	*β*	*T*	*p*	*R*	*R*^2^	*F*	*p*
HIV knowledge	.07	1.27	>.05	.77	.59	42.63	<.05
Attitudes to condom use	−.25	−4.02	<.05				
Vulnerability to HIV/AIDS	−.36	5.74	<.05				
Support to condom use	−.12	−2.01	<.05				
Condom use self-efficacy	−.29	−4.84	<.05				

Note: *p* < .5 level of significance.

Table 2 shows that HIV knowledge, attitude towards condom use, perceived vulnerability to HIV/AIDS, peer support to condom use, and condom use self-efficacy significantly jointly predicted sexual risk behaviours with *R* = .77, *R* ^2^ = .59, *F*(5, 148) = 42.63, and *p* < .05. This joint prediction accounted for 59% of the variance in sexual risk behaviours of the LDTDs. This result indicates a significant percentage of contribution of these psychological factors in explaining levels of sexual risk behaviours exhibited by drivers. In other words, some other psychological factors not investigated in the present study might have accounted for the 41% variation in sexual risk behaviour of the drivers.

In Table 2, the results show that attitude towards condom use (*β* = −.25; *t* = −4.02; *p* < .05), perceived vulnerability to HIV/AIDS (*β* = −.36; *t* = 5.74; *p* < .05), peer support to condom use (*β* = −.12; *t* = −2.01; *p* < .05), and condom use self-efficacy (*β* = −.29; *t* = −4.84; *p* < .05) significantly independently predicted sexual risk behaviours of LDTDs. Specifically, the result indicates that the more positive are attitudes of drivers towards condom use, the less their reported sexual risk behaviours. High perception of vulnerability to HIV/AIDS was negatively related to higher sexual risk behaviour among the drivers. This is interpreted that drivers who perceived themselves as more vulnerable to HIV/AIDS are likely to report lower sexual risk behaviours. A negative correlation was found between peer support to condom use and sexual risk behaviour. This implies that the more support drivers received from their peer on the use of condoms, the less likely they would engage in sexual risk behaviour. A negative correlation was also found between condom use self-efficacy and sexual risk behaviour. This result implies that drivers with high condom use self-efficacy reported lower sexual risk behaviours. However, HIV knowledge (*β* = .07; *t* = 1.27; *p* > .05) did not significantly independently predict sexual risk behaviours of drivers. This implies that HIV knowledge has no direct relationship with the level of sexual risk behaviours among the drivers. The hypothesis is therefore accepted.

*Second hypothesis:* Age, number of years of education, number of wives, number of intercourses in the last three months, number of partners apart from primary partners, and number of weeks spent outside home will independently and jointly significantly predict sexual risk behaviours among LDTDs. The result is presented in Table 3.

**Table 3.** Shows multiple regression of social factors predicting sexual risk behaviours among LDTDs.

Model	*β*	*T*	*p*	*R*	*R*^2^	*F*	*p*
Age of drivers	.17	1.79	>.05	.39	.15	4.39	<.05
Number of years of education	.36	4.53	<.05				
Number of wives	−.03	−0.32	>.05				
Number of intercourses in 3 months	.11	1.38	>.05				
Number of sexual partners	−.05	−0.06	>.05				
Weeks spent outside home	−.02	−0.19	>.05				

Note: *p* < .5 level of significance.

Table 3 shows that age, number of years of education, number of wives, number of intercourses in the last three months, number of partners apart from primary partners, and number of weeks spent outside home significantly jointly predicted sexual risk behaviour with *R* = .39, *R* ^2^ = .15, *F*(6, 147) = 4.39, and *p* < .05. This result indicates that all the social factors accounted for 15% of the variance in sexual risk behaviour among the drivers. It means that some other social factors not investigated in the present study might have accounted for the 85% variation in sexual risk behaviour of the drivers.

The result further shows that only number of years of education (*β* = .36; *t* = 4.53; *p* < .05) significantly independently predicted sexual risk behaviour among the drivers. This result shows that number of years of education has a positive relationship with sexual risk behaviour. This implies that the higher the number of years of education of drivers, the higher the level of sexual risk behaviour. However, age (*β* = .17; *t* = 1.79; *p* > .05), number of wives (*β* = −.03; *t* = 0.32; *p* > .05), number of intercourses in three months (*β* = .11; *t* = 1.38; *p* > .05), number of sexual partners (*β* = −.05; *t* = −0.06; *p* > .05), and number of times spent outside home (*β* = −.02; *t* = −0.19; *p* > .05) did not significantly independently predicted higher sexual risk behaviour among LDTDs. The hypothesis was not accepted.

## Discussion

We investigated HIV knowledge, attitudes towards condom use, perceived vulnerability to HIV/AIDS, perceived peer support to condom use, and condom use self-efficacy as psychological factors predicting sexual risk behaviour of LDTDs in a haulage company in Lagos, Nigeria. Also, the study investigated *t* age, number of years of education, number of wives, number of intercourses in the last three months, number of partners apart from primary partners, and number of weeks spent outside as social factors predicting sexual risk behaviour of drivers. Two hypotheses guided the study. The first hypothesis stated that HIV knowledge, attitude towards condom use, perceived vulnerability to HIV/AIDS, perceived peer support to condom use, and condom use self-efficacy would independently and jointly significantly predict sexual risk behaviour among LDTDs. All the psychological factors jointly predicted sexual risk behaviours of drivers. Interestingly, all the five psychological factors substantially contributed to the explanation of sexual risk behaviour of drivers. Apart from the joint contribution of the psychological factors, there were significant independent contributions of attitude towards condom use, perceived vulnerability to HIV/AIDS, perceived peer support to condom use, and condom use self-efficacy in levels of sexual risk behaviour of drivers. Specifically, it was found that drivers with positive attitudes towards condom use reported less sexual risk behaviour. The finding corroborates other previous studies that have confirmed the importance of truck drivers having positive attitudes towards condom use in relation to actually using condoms as a preventive measure during sex (Atilola et al., 2010; Matovu & Ssebadduka, 2014; Thakur, Toppo, & Lodha, 2015). This finding points out the need for truck drivers to understand the benefits of using condoms correctly and regularly, which may enable them to practise safer sexual behaviour and be sure of being free from any forms of sexually transmitted disease.

LDTDs who perceived themselves to be more vulnerable to HIV infection significantly reported less sexual risk behaviour. This finding is consistent with previous studies that have similarly reported a relationship between perception of vulnerability to HIV/AIDS and sexual risk behaviour of truck drivers (Atilola et al., 2010; Magno & Castellanos, 2016). The finding of our study suggests that how the drivers view themselves with regard to the possibility of being infected with HIV is very vital to their choice of sexual behaviour. More importantly in this study, perceived vulnerability to HIV/AIDS as one of the psychological factors investigated recorded the strongest contribution in explaining drivers’ sexual risk behaviour.

We also found that drivers who perceived higher support to the use of condom from their fellow drivers reported lower sexual risk behaviour. This finding corroborates the assertion made by Bandura (1994) about the behavioural benefits of conforming to social norms in an individual life. Thus, consistently yielding to the peer support to the regular use of condoms can increase the tendencies to engage in safer sexual behaviour by the drivers. Drivers with higher condom use self-efficacy reported lower sexual risk behaviour. The finding is in line with previous researchers who have reported the relevance of skill building such as condom use self-efficacy in HIV risk-reduction interventions (Lawal & Olley, 2015; O’Leary et al., 2008). Perhaps, the possibility of reduction in sexual risk behaviour due to higher condom use self-efficacy could be traced to the fact that truck drivers would have the ability to purchase condoms openly, negotiate with the sexual partner, and actually use the condoms.

HIV knowledge, however, did not have significant independent contributions to sexual risk behaviour of the drivers. This finding suggests that having the knowledge of HIV transmission alone may not make LDTDs to change their levels of sexual risk behaviour, but to know in addition to perceived vulnerability to HIV/AIDS, peer support to condom use, and condom use self-efficacy. The present finding is consistent with Sorensen et al. (2007) who found that HIV knowledge is inconsistent with condom use in the trucking population. In contrary, our finding did not support the previous studies that have showed a relationship between HIV knowledge and forms of sexual risk behaviours (Atilola et al., 2010; Awosan et al., 2014; Olugbenga-Bello et al., 2011). The present finding still suggests that changing sexual risk behaviour among the drivers should not be to increase their HIV knowledge alone, as this might not have much effect as found in the current study. Rather, an increase in HIV knowledge could be done along with condom attitudinal change and skill building in the use of condoms. In another perspective, LDTDs might be greatly aware of HIV/AIDS, but not accurately knowledgeable about HIV transmission and prevention.

The second hypothesis stated that age, number of years of education, number of wives, number of intercourses in the last three months, number of partners apart from primary partners, and number of weeks spent outside home would independently and jointly significantly predict sexual risk behaviour among LDTDs. All the social factors jointly predicted sexual risk behaviour of the drivers. This finding showed that all the social factors investigated in this study recorded a significant joint contribution to the levels of sexual risk behaviour among the drivers. The current finding is in line with previous studies that have identified social and structural factors to have a combined influence on sexual behaviours in the trucking population (Kohli et al., 2017; Sastry, 2016). For independent contribution of the social factors as predictor variables, it was revealed that only number of years of education independently predicted sexual risk behaviour among the drivers. It implies that drivers who might have spent more number of years in school education reported higher sexual risk behaviour. The current finding contradicts the previous study that gave mixed reports with no association between education and exposure to CSW, but with condom use (Chaturvedi et al., 2006). This previous finding throws light into why educated drivers in the current study reported higher sexual risk behaviour. It suggests that the educated truck drivers in this study might not have the comprehensive knowledge of HIV transmission and prevention that would guide them from engaging in risky sexual behaviour.

## Conclusion

Results of the present study demonstrate the relative importance of HIV knowledge, attitudes towards condom use, perceived vulnerability to HIV/AIDS, peer support to condom use, and condom use self-efficacy in explaining sexual risk behaviour of LDTDs. It can be concluded that all the psychological factors could jointly play important roles in the prevention intervention for reducing sexual risk behaviours of LDTDs. Of all these psychological predictor variables, only HIV knowledge had no significant independent contributions in predicting sexual risk behaviour of drivers. It is concluded that being knowledgeable about HIV alone may not be effective in reducing sexual risk behaviour of LDTDs; rather other predictor variables need to be added in the HIV risk-reduction intervention programmes. In addition, this finding might be an indication of incomprehensive knowledge of HIV transmission and prevention in the sampled truck drivers.

It was also found that all the social factors (age, number of years of education, number of wives, number of intercourses in the last three months, number of partners apart from primary partners, and number of weeks spent outside home) jointly predicted the sexual risk behaviour of LDTDs. This means that a combination of these social factors could play some role in explaining the practice of sexual risk behaviour among LDTDs. Similarly, all these social factors should be incorporated in prevention intervention programmes in changing or reducing sexual risk behaviours of LDTDs. However, only number of years of education was found to have a positive relationship with sexual risk behaviours among the drivers. It can be concluded that some drivers may be educated but still engage in some forms of sexual risk behaviours; therefore, preference should not be given alone to less-educated drivers as targets for HIV risk-reduction prevention interventions. Though other social factors had no independent relationships with sexual risk behaviours, their combined contributions appear to be relevant in HIV risk-reduction intervention programmes for the drivers.

## Implications and recommendations

Findings of this study imply that for a significant reduction in sexual risk behaviours of LDTDs, they need to possess a positive attitude towards condom use, support the use of condoms, see themselves as vulnerable to HIV infection, and be efficacious in the use of condoms. We therefore recommend that stakeholders at various settings including psychologists, health practitioners, governmental (National Agency for the Control of AIDS) and non-governmental agencies, religious bodies, and community leaders should all take the issue of high sexual risk behaviour in the trucking population as a matter of urgency. This is because truck drivers who do not admit to being vulnerable to HIV infection and do not view the condom-related variables investigated in this study as vital might easily engage in any form of risky sexual behaviour, putting their wives and other sexual partners at risk to the infection. It is also recommended that all the predictor variables should be considered in HIV prevention and risk-reduction interventions for LDTDs. Specifically, stakeholders and health educators should sensitise truck drivers on the need to realise that they are a high-risk group and are more vulnerable to HIV infection; thus, behavioural change is indispensable in their sexual relationships.

## Limitations of the study

This study faced some challenges that need to be put into consideration for future similar studies. Findings of the study were limited to LDTDs sampled from a single haulage company in Lagos. The inclusion of more haulage companies would help in generalisation of the findings. Another limitation is informant reliability – the subject matter is sensitive and controversial and informants may not be truthful for many reasons, including giving the ‘correct’ or ‘moral’ answer, or giving answers that reflect well on them or that they feel we expect.
